# Anxiety Status of Female Chinese Ph.D. Candidates and Its Association with Sports

**DOI:** 10.3390/healthcare10071203

**Published:** 2022-06-27

**Authors:** Yupeng Mao, Yongsheng Zhu, Changjun Jia, Fengxin Sun, Song Chen, Bing Liu

**Affiliations:** 1 Physical Education Department, Northeastern University, Shenyang 110819, China; maoyupeng@pe.neu.edu.cn (Y.M.); 2001276@stu.neu.edu.cn (Y.Z.); 2071367@stu.neu.edu.cn (C.J.); 2171435@stu.neu.edu.cn (F.S.); 2 School of Arts, Beijing Sport University, Beijing 100084, China

**Keywords:** sports, female Ph.D. candidates, anxiety

## Abstract

Given that stress leads to more anxiety among female Ph.D. candidates, more attention should be paid to their healthy lifestyle options. Several studies have shown that there is a negative correlation between sports and anxiety. This study took female Chinese Ph.D. candidates’ anxiety and sports participation as the research objects. A questionnaire entitled “Investigation on anxiety and sports of Ph.D. candidates” was used to explore the characteristics of anxiety in female Chinese Ph.D. candidates and to investigate the association between anxiety and sports in female Chinese Ph.D. candidates. A total of 588 Ph.D. candidates participated in the questionnaire survey. Some 21 invalid questionnaires were eliminated through the standard deviation of the items of the scale, and 567 valid questionnaires were finally obtained. The questionnaire survey was conducted online from 26 February to 15 March 2022, using the convenience sampling method. The results show that the anxiety level of female Chinese Ph.D. candidates is higher than that of male Ph.D. candidates and that the anxiety level of female Ph.D. candidates in a non-sports discipline is the highest. Weekly sports participation significantly lowers female Ph.D. candidates’ anxiety level (*p* < 0.01). Physical fatigue caused by study and work hinders female Ph.D. candidates from participating in sports (*p* < 0.05). Some female Chinese Ph.D. candidates in a negative emotional state are unwilling to participate in sports (*p* < 0.01). Future research should formulate different types of sports intervention programs suitable for alleviating the anxiety of female Ph.D. candidates.

## 1. Introduction

In recent years, the stress faced by Ph.D. candidates has attracted widespread attention in academia. A number of studies have shown that long-term investment in academic research makes graduate students, especially Ph.D. candidates, six times more likely to experience anxiety or depression than the general public, ref. [1] Ph.D. candidates frequently feel a sense of urgency, worry, and stress as they work toward their doctoral degrees. A total of 32% of Ph.D. candidates suffer from anxiety or are on the verge of mental problems [2]. More than one-third of Ph.D. candidates have varying degrees of anxiety [3]. In particular, the mental health of female Ph.D. candidates is worse than that of male Ph.D. candidates, especially with regard to depression and anxiety [4]. Brown and Watson interviewed eight female Ph.D. candidates in the UK and found that great pressure is caused by the problem of balancing family and academic life [5]. Haynes conducted a semi-structured interview with eight female Ph.D. candidates at a research university in the United States and found that they had problems with stress, social support, and self-esteem. This may be related to the role conflict caused by women’s multiple identities [6]. Some scholars have also examined the academic experience of female Ph.D. candidates majoring in science and engineering. Researchers have concluded that such candidates experience more difficulties than other disciplines due to cultural gender differences [7]. Sociodemographic variables, such as gender, age, marital status, motherhood, grade, and discipline have been associated with the anxiety of Ph.D. candidates [6,8]. Stress from learning, family, and employment leads to more anxiety among female Ph.D. candidates [6]. However, there have been few reports on the anxiety status of female Chinese Ph.D. candidates. Further exploration among female Chinese Ph.D. candidates is needed.

In recent years, several studies have shown that the effect of sports is not limited to physical health but can also eliminate negative emotions and improve psychological adaptability [9]. There is a negative correlation between sports and anxiety [10]. Empirical studies have shown that regular and appropriate participation in sports can reduce negative emotional experiences and reduce the adverse effects of negative emotions such as depression, anxiety, and stress [11,12]. Sports can enhance executive function [13] and conversion function [14], increase positive emotion, improve emotional regulation, and improve individual subjective evaluation of stress, thereby alleviating negative cognition and negative emotion and reducing the duration and degree that stress events have on individuals [15]. The research of Lane and Lovejoy showed that sports can have an impact on mood. After participating in a 60-min aerobic dance course, the subjects’ anger, confusion, fatigue, and tension were significantly reduced [16]. Some studies have shown that short-term aerobic exercise with autonomous intensity can alleviate anxiety better than resistance training [17,18]. Anxiety can be alleviated significantly by participating in moderate-intensity sports for 50 min, three times a week [19].

Studies show that Ph.D. candidates lack time and energy for leisure activities, including sports, due to their heavy learning schedules [6,20]. The interview research on the anxiety and stress of female Chinese Ph.D. candidates shows that some of them devote more time to scientific research to graduate on time than male Ph.D. candidates. Some of them are also responsible for childcare. They are, therefore, not willing to spend their time on leisure, sports, etc., even though they know that these activities may help them separate their attention from anxiety situations [21]. Studies found that men are more likely to participate in sports than women. The same situation also exists in college students [22].

In summary, sports play a role in alleviating anxiety. However, there has been little research and analysis of female Chinese Ph.D. candidates’ sports participation. In particular, there is a lack of in-depth studies on the relationship between sports and female Chinese Ph.D. candidates’ anxiety. Based on this, this study took anxiety and sports participation among Chinese Ph.D. candidates as the research object. A questionnaire was used to explore the characteristics of female Chinese Ph.D. candidates’ anxiety and to reveal the association between female Chinese Ph.D. candidates’ anxiety and sports. Consequently, this study proposes a hypothesis that there is a correlation between female Chinese Ph.D. candidates’ anxiety and sports. Therefore, there are four aspects of this hypothesis to be addressed. The first question involves the investigation of female Chinese Ph.D. candidates’ anxiety. The second question relates to the comparison of different groups of female Chinese Ph.D. candidates’ anxiety characteristics. The third aspect deals with the analysis of female Chinese Ph.D. candidates’ sports participation characteristics. Finally, the assessment of the association between sports and female Chinese Ph.D. candidates’ anxiety will be completed.

## 2. Methods

### 2.1. Participants

The questionnaire survey was conducted online from 26 February to 15 March 2022. To make the research easier to implement, a convenience sampling method was used. To improve the accuracy of the convenience sampling method, 13 universities with relatively complete Ph.D. programs were selected for sampling. We selected the 13 universities according to the type of university, reputation for academic rigor, and the scale of Ph.D. programs. From the perspective of university types, all these universities have Ph.D. programs, and the disciplines include humanities and social sciences, natural sciences and engineering, and technical sciences. Each of the universities has a large number of Ph.D. candidates. In terms of academic rigor, they include first-class universities and ordinary universities, which helps to reflect the anxiety and sports characteristics of Ph.D. candidates at differently ranked universities. The inclusion criteria of Ph.D. candidates are the registered Ph.D. candidates from the 13 universities. This study obtained the informed consent of all Ph.D. candidates participating in the survey. The way to obtain informed consent is to point out in the directions for the questionnaire that “If you are willing to accept the survey, please complete the questionnaire”. Our research was approved by the Biological and medical ethics committee of Northeastern University. The approval number is No.: 2022-04-117. Finally, a total of 588 Ph.D. candidates participated in the questionnaire survey. To improve the quality of the questionnaire, we used the descriptive analysis method to analyze each item in the scale. We set the value of data greater than ±3 standard deviation as an abnormal value and deleted it. Finally, 21 invalid questionnaires were found and deleted, and 567 valid questionnaires were finally obtained.

### 2.2. Instruments

The purpose of this study is to investigate the association between anxiety and sports participation in female Chinese Ph.D. candidates. A questionnaire entitled “Investigation on anxiety and sports of Ph.D. candidates” was used to explore the characteristics of anxiety in female Chinese Ph.D. candidates and to assess the association between sports participation and anxiety. The instruments included a sociodemographic questionnaire, a sports participation questionnaire, and the Spielberger State–Trait Anxiety Inventory. The sociodemographic questionnaire and sports participation questionnaire were investigated by us. We compiled the questionnaire items by reading the literature and consulting experts. The sociodemographic questionnaire provided information on gender, age, marital status, motherhood, grade, and discipline. The sports participation questionnaire provided information on sports behavior, sports motivation, and influencing factors of sports. The items of the two questionnaires were verified by consulting experts. The Spielberger State–Trait Anxiety Inventory (STAI) was used to investigate the anxiety of female Ph.D. candidates. The STAI is a self-report questionnaire that assesses anxiety as a state and as a trait. The State Anxiety Inventory (SAI) consists of 20 items rated on a four-point Likert scale ranging from 1 (“not at all”) to 4 (“very much”). The Trait Anxiety Inventory (TAI) consists of 20 items rated on a four-point Likert scale ranging from 1 (“almost never”) to 4 (“almost always”). Participants were asked to rate the statements according to how they currently feel (state) or how they feel in general (trait). Scores for both subscales have a range of 20–80. The SAI measures an individual’s transitory emotional response to a stressful situation. It evaluates the emotional response of worry, nervousness, tension, and feelings of apprehension related to how people feel “right now” in a stressful situation. The TAI measures an individual’s predisposition to anxiety determined by his/her personality and estimates how a person feels generally. The SAI and the TAI have well-established criterion, construct validity, and internal consistency reliability coefficients [23,24]. Li Wenli and Qian Mingyi translated the scale into Chinese and confirmed the validity of the instrument. The internal consistency of the Chinese state (Cronbach’s alpha = 0.91) and trait (Cronbach’s alpha = 0.88) items were excellent [25]. Many studies have shown that the STAI has good reliability and validity. Given the preestablished psychometric properties of the survey tools, construct validity and reliability diagnostics were not performed for this study.

### 2.3. Procedures

The main variables and their operationalization are summarized in Table 1. The dependent variable of the study is the Ph.D. candidates’ anxiety, including state anxiety and trait anxiety. The Ph.D. candidates were asked about their perception of state and trait anxiety in the items of the STAI. They were asked to choose the items in the SAI according to their most appropriate feeling at the moment and answer in the four grades of “Not at all”, “Somewhat”, “Moderately”, and “Very much” and choose the items in the TAI according to their most appropriate feeling at the moment and answer in the four grades of “Almost never”, “Somewhat”, “Always”, and “Almost always”. The independent variables include two dimensions: individual characteristics and sports characteristics. The individual characteristics dimension includes gender, age, marital status, motherhood, grade, and discipline category. The sports characteristics dimension includes three indexes: sports behavior, sports motivation, and sports influencing factors.

### 2.4. Statistical Analysis

SPSS 21.0 statistical software was used to analyze the data. The frequency analysis was used to analyze the individual characteristics and sports characteristics of Ph.D. candidates. A correlation test was used to analyze the individual characteristics and sports characteristics related to the anxiety of Ph.D. candidates. A regression test was used to analyze the independent variables related to Ph.D. candidates’ anxiety. Ph.D. candidates’ anxiety was measured by a comparison test to analyze the independent variables with linear correlation with candidates’ anxiety and the effects of marital status and motherhood on that anxiety.

## 3. Results

### 3.1. Basic Information of Samples

A total of 567 Ph.D. candidates were included in this study, including 247 male Ph.D. candidates and 320 female Ph.D. candidates. Some 56.44% of the Ph.D. candidates were between 26 and 30 years old, and 22.93% of them were between 31 and 35 years old. The proportion of unmarried Ph.D. candidates was higher than that of married Ph.D. candidates, which was 68.78%. The proportion of childless Ph.D. candidates was higher than that of Ph.D. candidates with children, and its proportion was 80.07%. The grade distribution of Ph.D. candidates was relatively uniform. In terms of disciplines, 35.63% of them came from physical education, and other disciplines were evenly distributed. The basic information of samples is shown in Table 2.

### 3.2. Analysis of Female Chinese Ph.D. Candidates’ Anxiety

#### 3.2.1. The Anxiety Level of Female Ph.D. Candidates in Non-Sports Disciplines Is the Highest

The Ph.D. candidates’ anxiety scores in different groups are shown in Table 3. The results show that the average scores of state anxiety and trait anxiety of female Ph.D. candidates in a non-sports discipline are the highest. The average score of trait anxiety of female Ph.D. candidates is higher than that of male Ph.D. candidates. The average scores of state anxiety and trait anxiety of female Ph.D. candidates in a sports discipline are the lowest. This study also found that the average score of state anxiety of male Ph.D. candidates is higher than that of female Ph.D. candidates, and the average score of state anxiety of male Ph.D. candidates in a sports discipline is the highest. The average score of trait anxiety of male Ph.D. candidates is lower than that of female Ph.D. candidates.

#### 3.2.2. Grade Positively Affects the Anxiety Level of Female Chinese Ph.D. Candidates

**Figure 1:**
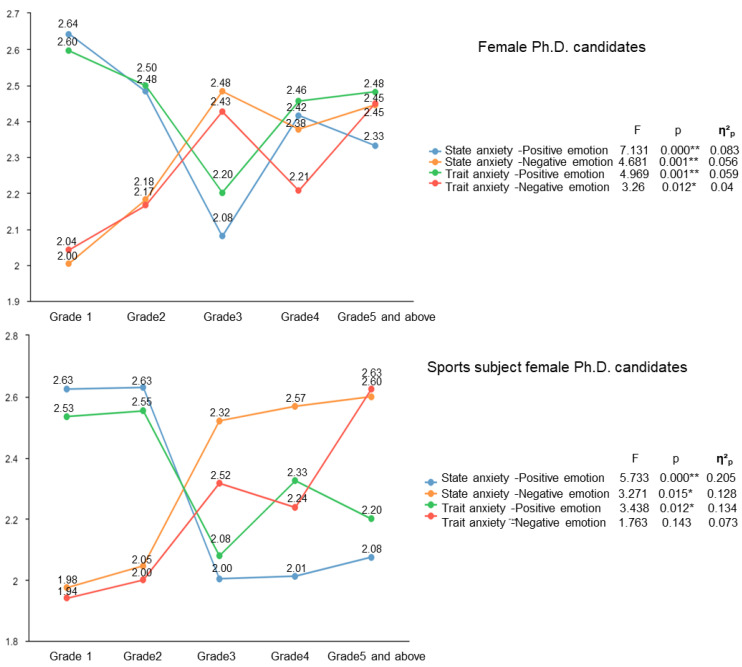
Effect of grade on anxiety of female Chinese Ph.D. candidates’ anxiety (* *p* < 0.05, ** *p* < 0.01).

Table 4 shows the relationship between different groups of female Chinese Ph.D. candidates’ anxiety and individual characteristics. The anxiety level of female Ph.D. candidates in a sport discipline has a linear correlation with grade. The numbers in the table are *p* values. Figure 1 shows that the positive emotions of the two groups decrease with the increase of grade, and their negative emotions increase with the increase of grade. As a result, the anxiety of the two groups increases with the increase of grade, and the anxiety level of Grade 3 is the highest. The state anxiety of female Ph.D. candidates in a non-sports discipline is also related to their grades (Table 4). The increase of grade leads to more negative emotions in a non-sports discipline, but there is no linear correlation between their grade and state anxiety.

#### 3.2.3. The Anxiety Level of Married Female Chinese Ph.D. Candidates with Children Is Higher Than That of Unmarried and Childless Ones

To further analyze the impacts of marital status and motherhood on Ph.D. candidates, we took the individual characteristics and sports characteristics related to their anxiety as covariates to investigate the impacts of marital status and motherhood on their anxiety (Table 5). The results show that the anxiety level of married female Ph.D. candidates is significantly higher than that of unmarried ones, and the anxiety level of married female Ph.D. candidates with children is the highest, but this result is not seen in male Ph.D. candidates. The anxiety level of married female Ph.D. candidates in a sports discipline is significantly higher than that of unmarried ones, but this result is not seen in male Ph.D. candidates in a sports discipline. The anxiety level of Ph.D. candidates with children in a sports discipline is significantly higher than that of the childless ones. However, the anxiety level of married female Ph.D. candidates with children in a non-sports discipline is significantly lower than that of unmarried ones.

### 3.3. Sports Characteristics of Female Chinese Ph.D. Candidates

#### 3.3.1. Weekly Sports Participation Frequency and Duration of Female Chinese Ph.D. Candidates

**Figure 2:**
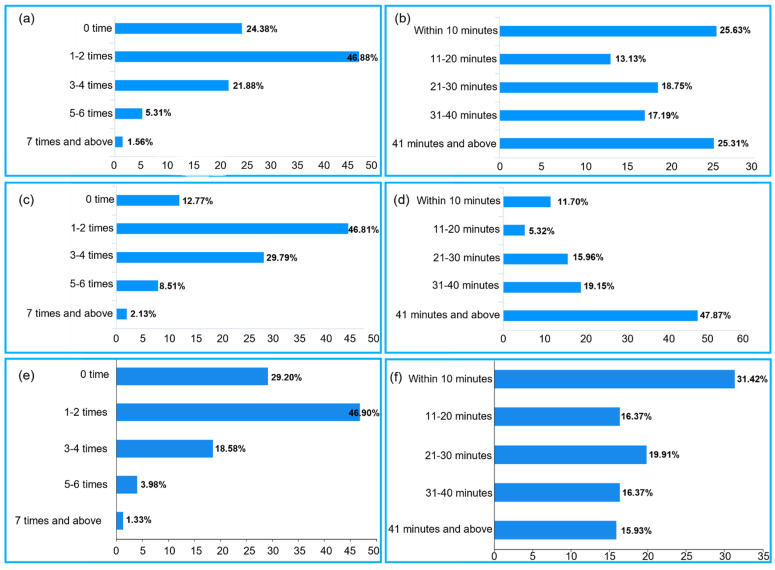
Weekly sports participation frequency and duration of female Chinese Ph.D. candidates: (**a**) weekly sports participation frequency of female Ph.D. candidates overall; (**b**) duration of female Ph.D. candidates overall; (**c**) weekly sports participation frequency of female Ph.D. candidates in a sports discipline; (**d**) duration of female Ph.D. candidates in a sports discipline; (**e**) weekly sports participation frequency of female Ph.D. candidates in a non-sports discipline; (**f**) duration of female Ph.D. candidates in a non-sports discipline.

The proportion of overall female Ph.D. candidates who often participate in sports is 29%. The proportion of their participation in sports 1–2 times a week is 47%, and 24% of them never participate in sports (Figure 2a). The duration of their participation in sports is mainly under 10 min or more than 41 min (Figure 2b). Figure 2c shows the weekly sports frequency of female Ph.D. candidates in a sports discipline. The results show that about 40% of them often participate in sports, 47% of them participates in sports 1–2 times a week, and 13% of them never participate in sports. Figure 2d shows the duration of female Ph.D. candidates in a sports discipline. The duration of their participation in sports is mainly more than 41 min, 35% of them participate in sports for 21–40 min each time, and only 17% of them participate in sports for 20 min each time. Figure 2e shows the weekly sports frequency of female Ph.D. candidates in a non-sports discipline. The results show that only 24% of them often participate in sports, 47% of them participate in sports 1–2 times a week, and 29% of them never participate in sports. Figure 2f shows the duration of exercise in female Ph.D. candidates in a non-sports discipline. The duration of their participation is mainly under 30 min, 31% of them participates in sports under 10 min, and only 32.3% of them participate in sports for more than 30 min each time.

#### 3.3.2. Sports Motivation and Influencing Factors of Female Chinese Ph.D. Candidates

**Figure 3:**
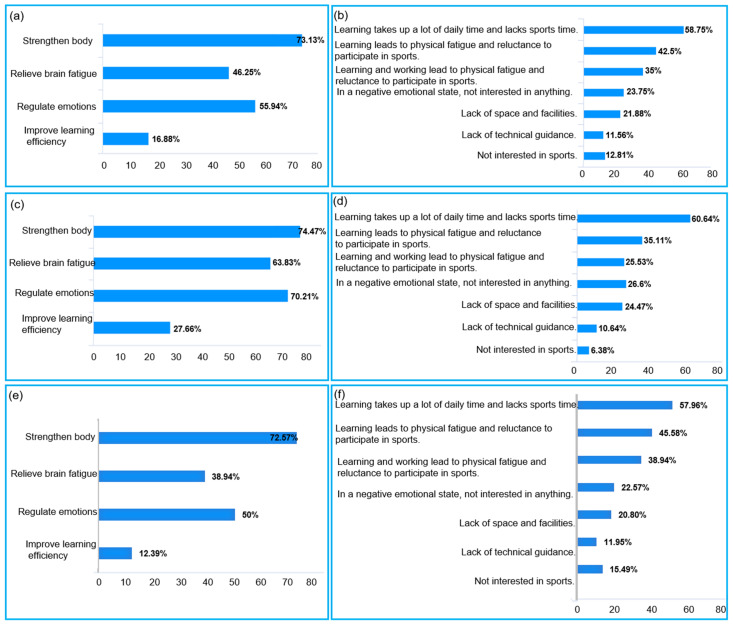
Sports motivation and influencing factors of female Chinese Ph.D. candidates: (**a**) sports motivation of female Ph.D. candidates overall; (**b**) influencing factors of female Ph.D. candidates overall; (**c**) sports motivation of female Ph.D. candidates in a sports discipline; (**d**) influencing factors of female Ph.D. candidates in a sports discipline; (**e**) sports motivation of female Ph.D. candidates in a non-sports discipline; (**f**) influencing factors of female Ph.D. candidates in a non-sports discipline.

Figure 3a shows that the sports motivation of female Ph.D. candidates’ overall participation in sports is mainly to strengthen their bodies and regulate their emotions. Figure 3b shows that the main factor affecting their participation in sports is the pressing learning task. Figure 3c shows that the motivation of female Ph.D. candidates in a sports discipline to participate in sports is mainly to strengthen their bodies, regulate their emotions, and alleviate brain fatigue. Figure 3d shows the main factor affecting their participation in sports is the pressing learning task. Figure 3e shows that the motivation of female Ph.D. candidates in a non-sports discipline to participate in sports is mainly to strengthen their bodies and regulate their emotions. Figure 3f shows the main factor affecting their participation in sports is the pressing learning task. Learning and working lead to their physical fatigue, which also affects their sports participation.

### 3.4. Relationship between Sports and Female Chinese Ph.D. Candidates’ Anxiety

#### 3.4.1. Sports Behavior Plays an Important Role in the Anxiety Level of Female Chinese Ph.D. Candidates

**Figure 4:**
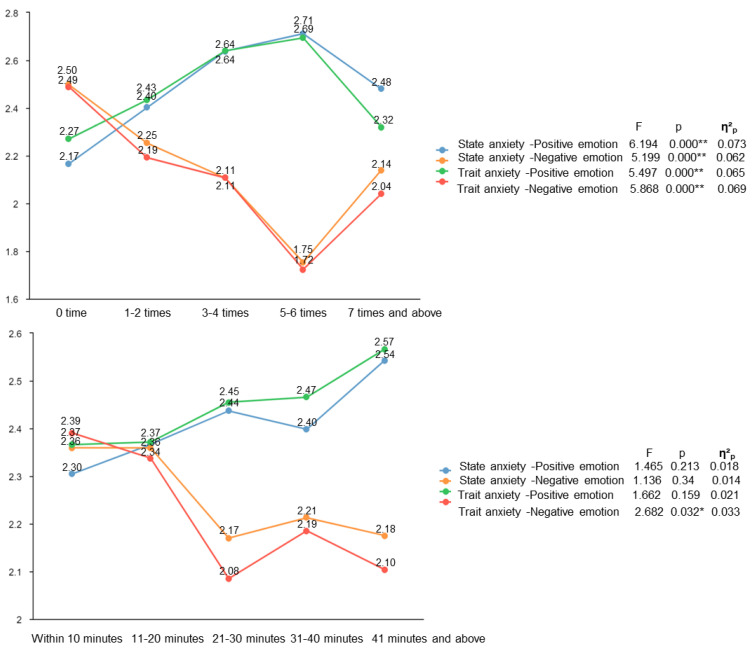
Effects of weekly sports participation frequency and duration on female Chinese Ph.D. candidates’ anxiety (* *p* < 0.05, ** *p* < 0.01).

The anxiety of female Ph.D. candidates is related to their sports participation (Table 6). Figure 4 shows that the frequency and duration of weekly sports participation significantly lowers anxiety, although the effect of duration is not significant.

#### 3.4.2. Differences in the Association between Anxiety and Sports Motivation among Female Chinese Ph.D. Candidates

Through correlation analysis, we found that the anxiety level of female Ph.D. candidates was related to their motivation to regulate emotions. Female Ph.D. candidates’ anxiety level correlated with emotional regulation motivation is high, but it is not significant. Table 7 shows the further one-way ANOVA coordinating anxiety and the motivation for regulating emotions in female Ph.D. candidates. The results show that the *p* value of the negative emotion of state anxiety is less than 0.05, which indicates that there are significant differences in the motivation of regulating emotions among the negative emotions of different state anxiety. The *p* value of the positive emotion of female Ph.D. candidates’ state anxiety in a non-sports discipline is less than 0.05, which means that there is a significant difference in the motivation of strengthening body among different positive emotions of state anxiety. The *p* value of positive and negative emotions of trait anxiety is less than 0.01, which means that there are extremely significant differences in strengthening body motivation among different trait anxiety emotions.

#### 3.4.3. Differences in the Association between Anxiety and Sports Participation Influencing Factors among Female Chinese Ph.D. Candidates

Through correlation analysis, we found that learning leads to physical fatigue in female Chinese Ph.D. candidates’ and makes them unwilling to participate in sports. Some of the female Chinese Ph.D. candidates in a negative emotional state are not interested in anything, significantly affecting their anxiety level. Specifically, candidates who are physically tired and in negative emotions due to learning are unwilling to participate in sports. At the same time, their negative emotion level is high. Table 8 is the further one-way ANOVA of anxiety and sports participation influencing factors of female Chinese Ph.D. candidates. The results show that the *p* value of the two influencing factors are both less than 0.05, which means that there are significant differences in the two influencing factors among different anxieties.

## 4. Discussion

This study found that female Chinese Ph.D. candidates’ trait anxiety level is higher than that of male Ph.D. candidates, which is consistent with the previous research results. According to the anxiety theory, anxiety is related to a disorder in individual emotion regulation and cognitive control. Studies have shown that women are more likely than men to be diagnosed with depression [26] and all anxiety disorders except obsessive–compulsive disorder [8]. It may be related to the fact that women are more vulnerable to negative emotional events [27,28] and have stronger susceptibility to negative emotions [29]. This is also the key reason for the high incidence of emotional disorders in women [30].

The results also indicated that grade has a significant effect on the anxiety of female Chinese Ph.D. candidates. Performing experiments, writing a doctoral thesis, and publishing doctoral qualification papers require considerable time, energy, and financial resources [31]. With the increase of grades, if Ph.D. candidates want to graduate on time, they will have less time to complete their studies and more stress and anxiety in the face of graduation and employment. As such, grade positively affects the anxiety level of female Chinese Ph.D. candidates.

The anxiety level of married and female Chinese Ph.D. candidates with children is significantly higher than that of unmarried and childless ones. However, this result is not seen in male Ph.D. candidates. Previous studies have shown that the stress of female Ph.D. candidates is related to the role conflict caused by women’s multiple identities [6]. While working hard for a doctorate, female Ph.D. candidates also spend time and energy taking care of their families as wives or mothers. It is a kind of stress for them, which leads to more anxiety.

Most importantly, this study found that weekly sports participation frequency and the duration of the participation lower anxiety levels. Those who participate in sports 5–6 times a week have the highest positive emotion and the lowest negative emotion. It is consistent with the results of many previous studies [11,12,32,33] that show sports can alleviate anxiety. Our conclusion that sports can reduce the female Chinese Ph.D. candidates’ anxiety level may be related to two results. First, previous studies have shown that sports can enhance executive [13] and conversion functions [14]. In this study, we imply that female Chinese Ph.D. candidates’ participation in sports makes them focus on sports and temporarily shift away from the stress situation. It may be one way to alleviate their anxiety. Second, sports can enhance positive emotional experiences, improve individual negative cognition and decrease negative emotions, reduce the duration and degree of the impact of stress [15], and help individuals face stress and difficulties more easily [34]. In addition, this study found that the female Chinese Ph.D. candidates in a non-sports discipline have anxiety levels higher than those in a sports discipline. We believe that participating in more sports reduces the anxiety of female Chinese Ph.D. candidates in a sports discipline. At the same time, the research results on the sports behavior also show that female Chinese Ph.D. candidates in a sports discipline often take part in sports, and the non-sports discipline’s ones are the opposite. It can be seen that those sports have a negative impact on female Chinese Ph.D. candidates’ anxiety. The above results suggest that participating in sports may help female Ph.D. candidates establish healthy lifestyles and is an effective way to reduce their anxiety.

Regulating emotions is the main sports motivations of female Chinese Ph.D. candidates. Emotions of Ph.D. candidates may be accompanied by caution, complaints, meditation, nervousness, and worry [35]. It may be due to Ph.D. candidates not only completing the doctoral program itself, but also balancing family and work. Particularly, female Chinese Ph.D. candidates face more role conflicts, such as, pregnancy and raising children [5]. Too much stress and role conflict make them more anxious. Therefore, they hope to regulate their emotions by participating in sports.

Finally, it has been shown that physical fatigue caused by learning and working is the main factor hindering female Chinese Ph.D. candidates from participating in sports. Some of the female Chinese Ph.D. candidates in negative emotional states are unwilling to participate in sports, and their anxiety level is high. Research has shown that participating in projects and publishing papers occupy most of a Ph.D. candidates’ daily life and are the main pressure during their learning career [1,36,37,38,39]. Therefore, female Chinese Ph.D. candidates’ lack of sports and unwillingness to spend time participating in sports may be related to their pressing learning task.

## 5. Conclusions

The strengths of this study are that statistical analysis is appropriate to access a wide sample of Chinese Ph.D. candidates. The results clearly show that the anxiety level of female Chinese Ph.D. candidates is higher than that of male Ph.D. candidates, and the anxiety level of female Chinese Ph.D. candidates in a non-sports discipline is the highest. In particular, the study provides quantified evidence of how sports participation lowers anxiety in female Chinese Ph.D. candidates, weekly sports participation significantly lowers anxiety level and physical fatigue caused by learning and working. It is this fatigue that is the main factor hindering female Chinese Ph.D. candidates from participating in sports.

Limitations of this study include the sampling and the use of only a self-report measure to evaluate anxiety. First, all participants were recruited from 13 universities in China, and the convenient sampling method was adopted to conduct the questionnaire survey, which may inevitably lead to the concentration of Ph.D. candidates in certain areas. Thus, lack of diverse backgrounds means these findings cannot be representative of all Ph.D. candidates in China. Second, the self-report scale has its own defects in that everyone may have their own response style. For example, some Ph.D. candidates like to use the extreme option “Very much” to describe their currently feelings, while some like to boast of rationality and objectivity, and always only choose the middle option “Moderately”. Fortunately, the STAI has designed reverse scoring items to control statistics, so future research suggests adding a rating scale or measuring at different time points.

This study agrees with the previous studies that sports can decrease anxiety. Future research should formulate different types of sports intervention schemes suitable for alleviating female Ph.D. candidates’ anxiety. In addition, it is also important to research the mitigation mechanism of different sports intervention schemes on female Ph.D. candidates’ anxiety.

## Figures and Tables

**Table 1:** Variables and their operationalization.

Variable Type		Variable Name	Operationalization
Dependent variable	Anxiety type	State anxiety	1 = Not at all, 2 = Somewhat, 3 = Moderately, 4 = Very much
		Trait anxiety	1 = Almost never, 2 = Somewhat, 3 = Always, 4 = Almost always
Independent variable	Individual characteristics	Gender	Male = 0, Female = 1
		Age	20–25 years old = 1, 26–30 years old = 2, 31–35 years old = 3, 36–40 years old = 4, 41 years old and above = 5
		Marital status	Unmarried = 0, Married = 1
		Motherhood	No = 0, Yes = 1
		Grade	Grade 1 = 1, Grade 2 = 2, Grade 3 = 3, Grade 4 = 4, Grade 5 and above = 5
		Discipline category	Natural science = 1, Medical Science = 2, Humanities and Social Sciences = 3, Agricultural Science = 4, Engineering and technical science = 5
		Sports discipline	No = 0, Yes = 1
	Sports characteristics	Sports behavior	Weekly sports frequency (0 time = 0, 1–2 times = 1, 3–4 times = 2, 5–6 times = 3, 7 times and above = 4)
			Duration of sports each time (Within 10 min = 0, 11–20 min = 1, 21–30 min = 2, 31–40 min = 3, More than 41 min = 4)
		Sports motivation (No = 0, Yes = 1)	Strengthen body
			Relieve brain fatigue
			Regulate emotions
			Improve learning efficiency
		Influencing factors of sports (No = 0, Yes = 1)	Learning takes up a lot of daily time and lacks sports time.
			Learning leads to physical fatigue and reluctance to participate in sports.
			Learning and working lead to physical fatigue and reluctance to participate in sports.
			In a negative emotional state, not interested in anything.
			Lack of space and facilities.
			Lack of technical guidance.
			Not interested in sports.

**Table 2:** Basic information of questionnaire survey samples.

**Essential Information**	**Classification Criteria**	**Frequency**	**Percentage (** **%** **)**
Gender	Male	247	43.56
	Female	320	56.44
Age	20–25 years old	72	12.7
	26–30 years old	320	56.44
	31–35 years old	130	22.93
	36–40 years old	27	4.76
	41 years old and above	18	3.17
Marital Status	Married	177	31.22
	Unmarried	390	68.78
Motherhood	Yes	113	19.93
	No	454	80.07
Grade	Grade 1	136	23.99
	Grade 2	200	35.27
	Grade 3	117	20.63
	Grade 4	68	11.99
	Grade 5 and above	46	8.11
Discipline	Natural science	101	17.81
	Medical Science	71	12.52
	Humanities and Social Sciences	242	42.68
	(Sports discipline)	(202)	(35.63)
	Agricultural Science	76	13.4
	Engineering and technical science	77	13.58

**Table 3:** Descriptive statistics of anxiety scores of different Chinese Ph.D. candidates.

Ph.D. Candidate Group	State Anxiety		Trait Anxiety	
	Male Average Value ± Standard Deviation	Female Average Value ± Standard Deviation	Male Average Value ± Standard Deviation	Female Average Value ± Standard Deviation
Overall	47.170 ± 10.26	46.663 ± 8.45	45.822 ± 8.12	46.697 ± 6.41
Sports discipline’s	47.194 ± 8.91	46.053 ± 6.33	45.796 ± 7.64	45.096 ± 6.95
Non-sports discipline’s	47.151 ± 9.32	46.916 ± 8.71	45.842 ± 8.73	47.363 ± 10.38

**Table 4:** Relationship between different groups of female Chinese Ph.D. candidates’ anxiety and individual characteristics.

Group	Essential Information	State Anxiety—Positive Emotion	State Anxiety—Negative Emotion	Trait Anxiety—Positive Emotion	Trait Anxiety—Negative Emotion
Overall	Age	−0.130 *	0.063	−0.1	0.046
	Marital status	−0.03	0.032	−0.037	0.069
	Motherhood	−0.053	0.013	−0.064	0.05
	Grade	−0.171 **	0.203 **	−0.113 *	0.149 **
	Discipline category	0.002	−0.089	0.051	−0.022
	Sports discipline	−0.073	0.011	−0.091	−0.076
Sports discipline	Age	−0.304 **	0.212 *	−0.238 *	0.188
	Marital status	−0.176	0.129	−0.206 *	0.263 *
	Motherhood	−0.156	0.119	−0.176	0.215 *
	Grade	−0.382 **	0.327 **	−0.242 *	0.240 *
Non-sports discipline	Age	−0.008	−0.014	0.01	0.024
	Marital status	0.071	−0.021	0.076	−0.001
	Motherhood	0.051	−0.067	0.048	−0.017
	Grade	−0.081	0.152 *	−0.06	0.115
	Discipline category	0.003	−0.106	0.061	−0.026

* *p* < 0.05, ** *p* < 0.01.

**Table 5:** Effect of marital status and motherhood of Chinese Ph.D. candidates’ anxiety.

Group	Gender	Marital Status & Motherhood Status		State Anxiety—Positive Emotion	State Anxiety—Negative Emotion	Trait Anxiety—Positive Emotion	Trait Anxiety—Negative Emotion
Overall	Female	Marital status	F	20.488	11.395	14.752	19.671
			*p*	<0.001 **	0.001 **	<0.001 **	<0.001 **
		Motherhood	F	16.483	27.232	19.041	7.164
			*p*	<0.001 **	<0.001 **	<0.001 **	0.001 **
	R^2^			0.778	0.784	0.729	0.740
	Male	Marital status	F	15.792	20.236	13.691	12.938
			*p*	<0.001 **	<0.001 **	<0.001 **	<0.001 **
		Motherhood	F	14.96	16.577	5.885	9.138
			*p*	<0.001 **	<0.001 **	<0.001 **	<0.001 **
		R^2^		0.823	0.826	0.786	0.802
Sports discipline	Female	Marital status	F	16.133	1.09	0.128	6.446
			*p*	<0.001 **	0.3	0.722	0.013 *
		Motherhood	F	10.833	11.543	1.683	0.021
			*p*	<0.001 **	<0.001 **	0.192	0.979
		R^2^		0.810	0.783	0.716	0.713
	Male	Marital status	F	6.932	2.476	14.354	21.613
			*p*	0.010 **	0.119	<0.001 **	<0.001 **
		Motherhood	F	8.497	3.421	4.089	10.305
			*p*	<0.001 **	0.037*	0.020*	<0.001 ***
		R^2^		0.861	0.838	0.821	0.850
Non-sports discipline	Female	Marital status	F	9.399	4.163	16.875	15.576
			*p*	0.002 **	0.043 *	<0.001 **	<0.001 **
		Motherhood	F	6.626	15.671	18.869	9.833
			*p*	0.002 **	<0.001 **	<0.001 **	<0.001 **
		R^2^		0.772	0.794	0.750	0.770
	Male	Marital status	F	13.494	10.687	1.516	3.233
			*p*	<0.001 **	0.001 **	0.22	0.075
		Motherhood	F	6.475	14.353	2.651	2.725
			*p*	0.002 **	<0.001 **	0.074	0.069
		R^2^		0.811	0.830	0.769	0.798

* *p*<0.05, ** *p*<0.01.

**Table 6:** Correlation between sports behavior and female Chinese Ph.D. candidates’ anxiety.

Group	Anxiety Type and Emotion Type	Weekly Sports Frequency	Duration of Sports
Overall	State anxiety—Positive emotion	0.248 **	0.126 *
	State anxiety—Negative emotion	−0.231 **	−0.103
	Trait anxiety—Positive emotion	0.216 **	0.138 *
	Trait anxiety—Negative emotion	−0.240 **	−0.156 **
Sports discipline	State anxiety—Positive emotion	0.337 **	0.083
	State anxiety—Negative emotion	−0.304 **	−0.045
	Trait anxiety—Positive emotion	0.368 **	0.143
	Trait anxiety—Negative emotion	−0.273 **	−0.023
Non-sports discipline	State anxiety—Positive emotion	0.238 **	0.195 **
	State anxiety—Negative emotion	−0.209 **	−0.143 *
	Trait anxiety—Positive emotion	0.185 **	0.198 **
	Trait anxiety—Negative emotion	−0.213 **	−0.182 **

* *p* < 0.05, ** *p* < 0.01.

**Table 7:** Differences in the association between anxiety and sports motivation among female Chinese Ph.D. candidates.

Sports Motivation		State Anxiety—Positive Emotion	State Anxiety—Negative Emotion	Trait Anxiety—Positive Emotion	Trait Anxiety—Negative Emotion
Female Chinese Ph.D. candidates					
Regulating emotions	F	2.612	4.251	2.085	0.349
	*p*	0.107	0.040 *	0.15	0.555
	η²_p_	0.008	0.013	0.007	0.001
Female Chinese Ph.D. candidates in non-sports discipline.					
Strengthening body	F	6.031	3.562	11.332	10.054
	*p*	0.015 *	0.06	0.001 **	0.002 **
	η²_p_	0.026	0.016	0.048	0.043

* *p* < 0.05, ** *p* < 0.01.

**Table 8:** Differences in the association between anxiety and sports participation influencing factors among female Chinese Ph.D. candidates.

Influencing Factors		State Anxiety—Positive Emotion	State Anxiety—Negative Emotion	Trait Anxiety—Positive Emotion	Trait Anxiety—Negative Emotion
Learning leads to physical fatigue and reluctance to participate in sports.	F	5.934	5.144	5.553	10.938
	*p*	0.015 *	0.024 *	0.019 *	0.001 **
	η²_p_	0.018	0.016	0.017	0.033
In a negative emotional state, not interested in anything.	F	32.566	40.433	14.559	36.13
	*p*	<0.001 **	<0.001 **	<0.001 **	<0.001 **
	η²_p_	0.093	0.113	0.044	0.102

* *p* < 0.05, ** *p* < 0.01.

## Data Availability

The data presented in this study are available on request from the corresponding author.
